# Sponge diversity in Eastern Tropical Pacific coral reefs: an interoceanic comparison

**DOI:** 10.1038/s41598-019-45834-4

**Published:** 2019-06-28

**Authors:** José Luis Carballo, José Antonio Cruz-Barraza, Cristina Vega, Héctor Nava, María del Carmen Chávez-Fuentes

**Affiliations:** 1 Instituto de Ciencias del Mar y Limnología, Universidad Nacional Autónoma de México (Unidad Académica Mazatlán), Avenida Joel Montes Camarena s/n, Mazatlán (SIN) 82000, PO box 811, Mazatlán, Mexico; 2 Laboratorio de Biodiversidad Marina, Departamento de Zoología, Instituto de Investigaciones sobre los Recursos Naturales, Universidad Michoacana de San Nicolás de Hidalgo, Morelia (MICH), Mexico

**Keywords:** Biodiversity, Ocean sciences, Zoology

## Abstract

Sponges are an important component of coral reef communities. The present study is the first devoted exclusively to coral reef sponges from Eastern Tropical Pacific (ETP). Eighty-seven species were found, with assemblages dominated by very small cryptic patches and boring sponges such as *Cliona vermifera*; the most common species in ETP reefs. We compared the sponge patterns from ETP reefs, Caribbean reefs (CR) and West Pacific reefs (WPR), and all have in common that very few species dominate the sponge assemblages. However, they are massive or large sun exposed sponges in CR and WPR, and small encrusting and boring cryptic species in ETP. At a similar depth, CR and WPR had seven times more individuals per m^2^, and between four (CR) and five times (WPR) more species per m^2^ than ETP. Perturbation, at local and large scale, rather than biological factors, seems to explain the low prevalence and characteristics of sponge assemblages in ETP reefs, which are very frequently located in shallow water where excessive turbulence, abrasion and high levels of damaging light occur. Other factors such as the recurrence of large-scale phenomena (mainly El Niño events), age of the reef (younger in ETP), isolation (higher in ETP), difficulty to gain recruits from distant areas (higher in ETP), are responsible for shaping ETP sponge communities. Such great differences in sponge fauna between the three basins might have consequences for coral reef structure and dynamics.

## Introduction

Coral reefs are among the most complex, and largest biological structures on earth^1,2^, and probably are the most diverse communities of the oceans and have an estimated of 1,330,000 species^3,4^. They are considered the ‘ocean’s rainforest by their high productivity^5^, and although they occupy less than one percent of the Earth’s marine environment^6^, provide important services to human communities, which represent a value of 352,249 US$ ha^−1^year^−1^ ^7^.

In Eastern Tropical Pacific region (ETP), modern reef-building corals extend from the Gulf of California (Mexico) to Ecuador, with a distribution skewed toward the northern hemisphere^8,9^, since 46% of the coverage is located in Mexico^10^. These reefs are relatively recent, varying from 200 to 5600 years in age, with a thickness ranging from 0.2 to 13 m, 4.5 m in average^11^. They are mainly made up of interlocking, branching pocilloporids, constructed by very few species of the genus *Pocillopora*, or rarely built by massive corals of the genera *Porites* and *Pavona*^11,12^.

The general physical conditions of this vast region are not conducive to reef growth (see Discussion), and ETP reefs are typically small (a few hectares or less), patchily distributed, shallow and low in species diversity^13–15^. Comparing with Caribbean, ETP reefs are less vertically developed, less consolidated, smaller in extent and lacking biotic cementing and binding agents^16–18^.

In ETP there are also areas where corals do not form a continuous framework structure; rather, they form isolated patches of coral heads growing directly on bedrock called coral communities^17,19^. In both cases, whether forming true reefs or isolated patches, the interlocking, branching pocilloporids form a 3D framework with an extraordinary diversity of habitats.

The studies of coral reef organisms in ETP and their taxonomy are strong biased towards the most conspicuous organisms, such as fishes^20,21^, whereas most of the cryptic habitats that form the 3D framework remain largely ignored^18,22,23^. In fact, the coral reef cryptofauna community is understudied relative to surface reef fauna worldwide^23–25^.

Therefore, it is of great concern that our knowledge of coral associated invertebrates is so limited, especially in light of severe and ongoing degradation of coral reef habitats.

Sessile groups such as sponges usually dominate cryptofauna in coral reefs^21,22^. They constitute an abundant and functionally important component of coral reef systems that perform many important functional roles^26,27^. Sponges are important mediators of reef productivity^28–30^ and can take up dissolved organic matter (DOM) to generate an outflow of particulate organic matter (POM). Such outflow feeds other invertebrates at basal and intermediate levels of the reef trophic chain, therefore contributing to the energy requirements of these ecosystems via a pathway defined as the ‘sponge loop’^30,31^. Sponges also provide microhabitats for various invertebrate species as well as some fishes enhancing biodiversity^26,32^, and harbors microbial symbionts that can contribute to reef productivity^33–35^. Therefore, that changes in their abundance and diversity have the potential to affect overall reef ecosystem functioning. In addition, the importance of sponges on coral reefs worldwide is attracting more attention as the relative abundance of reef-building corals has declined^36–38^.

Unfortunately, despite of the important roles commented above, the sponge fauna of the ETP is probably the least known globally, with the exception of boring sponges^39–45^, and some conspicuous species^46–48^.

In this study, we examined patterns of sponge biodiversity in coral reefs across the entire Mexican Pacific Ocean. We also explore the possible causes that explain the differences between ETP and Caribbean (CR) and West Pacific Reefs (WPR).

## Material and Methods

### Study area

**Figure 1:**
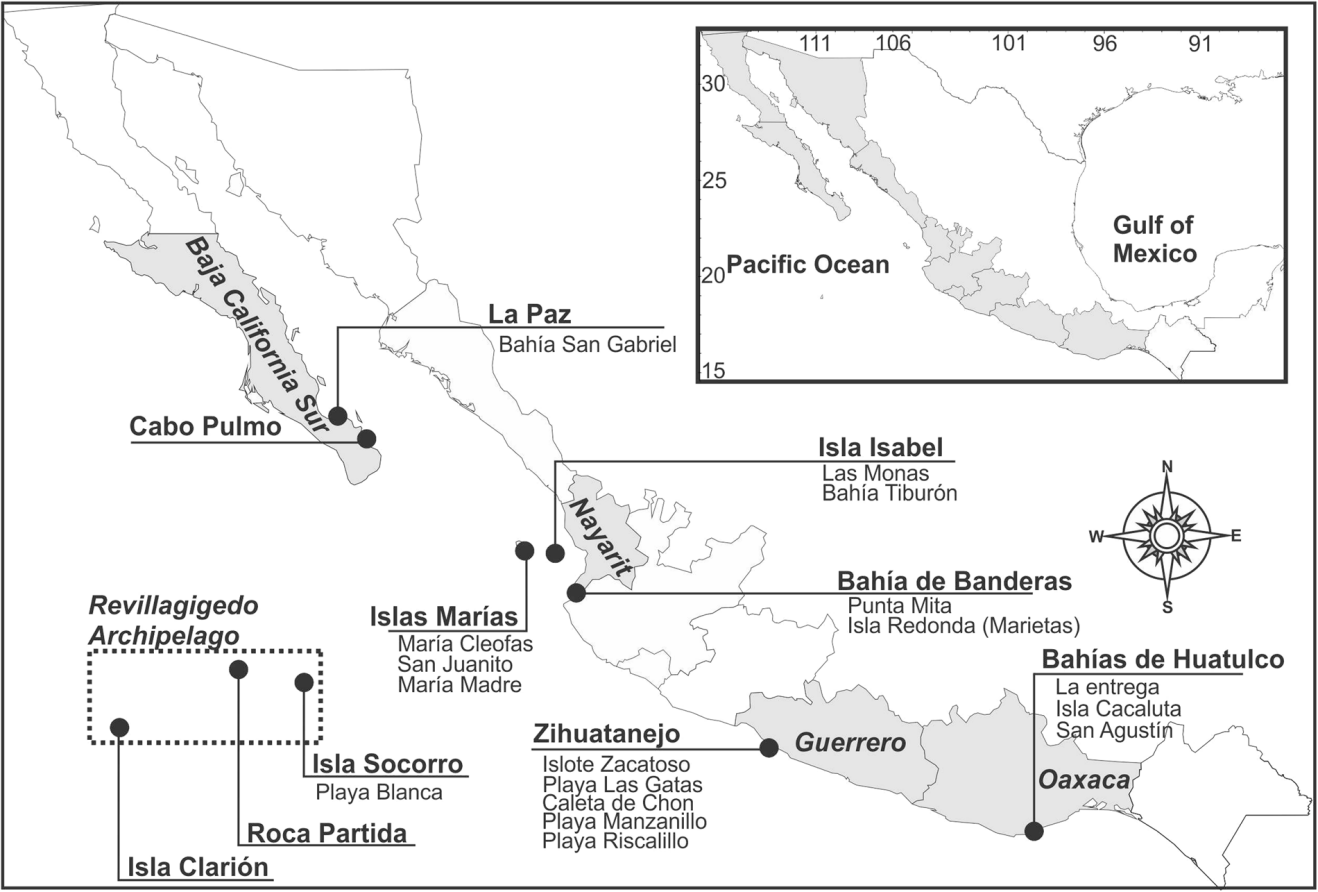
Sampling locations along the Pacific coast of Mexico.

**Figure 2:**
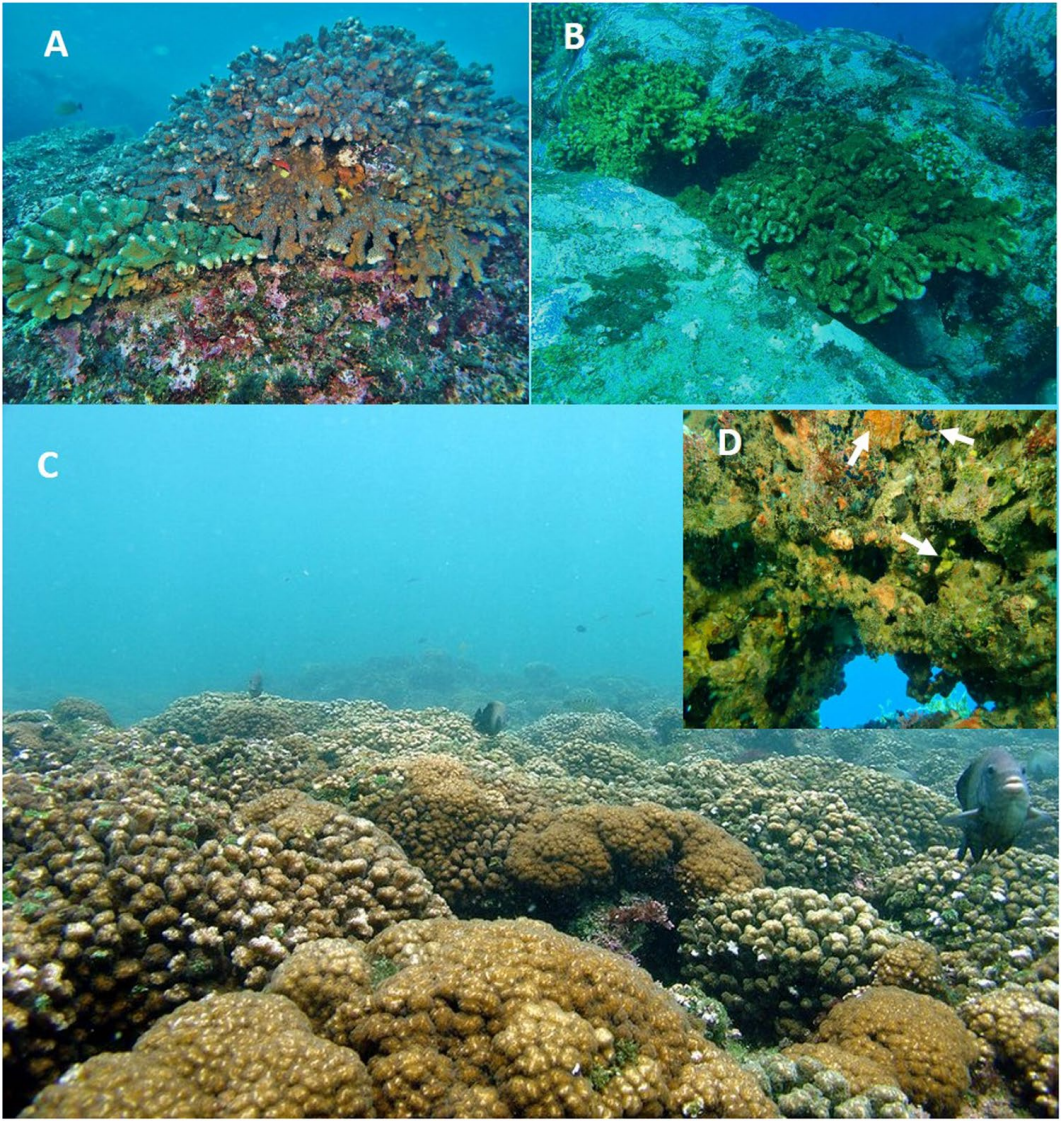
In ETP we can find two different reef formations: Coral communities, defined as areas with scattered colonies or isolated patches growing directly on bedrock (**A**,**B**), and coral reefs, when a reef framework is developed (**C**). (**D**), Lower surface of B, showing different species of cryptic sponges (arrows). Images taken by JLC.

The present study was carried out on 20 coral reefs and coral communities distributed along the Mexican Pacific coast (Fig. 1). We consider coral reefs as coral-built structures elevated above the bottom and developed over an accumulation of dead coral framework^49^ (Fig. 2). On the other hand, we define, coral communities as areas with scattered colonies or isolated patches growing directly on bedrock. There is no continuous framework and these areas tend to have a low percentage of coral cover^50^.

Owing to differences in the biological characteristics and composition of the coral communities, the reefs of the Pacific coast of México are naturally divided into three groups: those of the Gulf of California, the Revillagigedo Archipelago, and the tropical Mexican Pacific (Table 1).

**Table 1:** Presence or absence list of species in the three main coral reefs areas in Mexican Pacific.

Species	Gulf of California	Tropical Mexican Pacific	Revillagigedo Archipelago	Number of reefs	Frequency of occurrence (%)
*Acanthancora sp*.		X		2	10
*Acarnus erithacus*			X	1	5
*Acarnus oaxaquensis*		X		1	5
*Amphimedon texotli*		X		5	25
*Aplysilla glacialis*		X	X	5	25
*Aplysilla sulphurea*		X		1	5
*Aplysina clathrata*		X		1	5
*Aplysina gerardogreeni*	X	X		6	30
*Aplysina revillagigedi*			X	3	15
*Aplysina azteca*		X		1	5
*Aplysina sp*.		X		1	5
*Axinella nayaritensis*		X		1	5
*Batzella* sp. 1		X		1	5
*Callyspongia acapulcaensis*		X		3	15
*Callyspongia californica*	X	X		11	55
*Chalinula nematifera*	X	X		4	20
*Chelonaplysilla violacea*		X	X	7	35
*Chondrilla montanusa*		X		1	5
*Chondrilla pacifica*	X		X	2	10
*Chondrosia tenochca*		X		4	20
*Cinacyrella* sp.		X		1	5
*Cladocroce reina*		X		2	10
*Clathria (Microciona)* sp. 1		X		1	5
*Clathria (Microciona)* sp. 2		X		1	5
*Clathria (Microciona)* sp. 3		X		1	5
*Clathria (Thalysias)* sp 1.		X		1	5
*Cliona amplicavata*	X	X		12	60
*Cliona californiana*		X	X	2	10
*Cliona euryphylle*	X	X		6	30
*Cliona flavifodina*	X	X	X	5	25
*Cliona medinae*			X	2	10
*Cliona microstrongylata*	X			2	10
*Cliona mucronata*	X	X	X	10	50
*Cliona pocillopora*	X	X	X	9	45
*Cliona raromicrosclera*	X			1	5
*Cliona* sp.		X		3	15
*Cliona tropicalis*	X	X	X	14	70
*Cliona vallartense*	X	X		3	15
*Cliona vermifera*	X	X	X	18	90
*Cliothosa tylostrongilata*		X		1	5
*Desmanthus levii*		X		1	5
Dyctioceratida indt.		X		2	10
*Erylus sollasii*			X	2	10
*Geodia media*	X	X	X	4	20
*Haliclona caerulea*		X		9	45
*Haliclona* sp.1		X		2	10
*Haliclona* sp. 2		X		1	5
*Haliclona* sp. 3			X	1	5
*Haliclona turquoisia*		X		2	10
*Halicnemia diazae*		X	X	3	15
*Halisarca sacra*		X		1	5
*Hexadella pleochromata*		X		3	15
*Hyattella intestinalis*		X		3	15
*Hymeniacidon* sp.		X		1	5
*Ircinia* sp.		X		4	20
*Lissodendoryx albemarlensis*		X		1	5
*Lissodendoryx schmidti*		X		2	10
*Megaciella toxispinosa*	X			1	5
*Mycale cecilia*		X	X	10	50
*Mycale magnirhaphidifera*		X		8	40
*Mycale magnitoxa*		X	X	4	20
*Penares cortius*		X		1	5
*Pione carpenteri*	X	X	X	15	75
*Pione mazatlanensis*	X	X		6	30
*Placospongia carinata*		X		1	5
*Plakina muricyae*		X	X	4	20
*Plakina paradilopha*			X	1	5
*Plakortis albicans*		X	X	4	20
*Plakortis clarionensis*			X	1	5
*Prosuberites psamophillus*		X	X	4	20
*Pseudosuberites* sp.		X		1	5
*Scopalina ruetzleri*		X		5	25
*Siphonodictyon crypticum*		X	X	11	55
*Spheciospongia incrustans*		X		4	20
*Spheciospongia ruetzleri*		X		1	5
*Spirastrella decumbens*		X	X	2	10
*Stoeba* sp.		X		2	10
*Strongylacidon* sp.			X	1	5
*Suberea etiennei*		X		2	10
*Tedania tropicalis*		X		4	20
*Terpios sp*.		X		1	5
*Thoosa calpulli*	X	X	X	13	65
*Thoosa mismalolli*	X	X	X	15	75
*Thoosa purpurea*			X	1	5
*Thoosa* sp.		X		3	15
*Timea chiasterina*		X		1	5
*Timea juantotoi*		X		1	5
TOTAL SPECIES PER REGION	20	74	30		

The fourth column (Number of reefs) shows the number of reefs in which appears each species. The fifth column shows the frequency of occurrence (i.e., the percent of reefs in which the species was found). Species are arranged alphabetically.

The most important coral reefs in the ETP are in the southern region of Mexico (tropical Mexican Pacific), which harbors some of the most developed reefs such as La Entrega and San Agustín (Oaxaca state), and Caleta de Chon, Playa Manzanillo and Islote Zacatoso (Guerrero state)^11,13,51^. Nayarit state, in the center of the region, harbored some of the most important coral communities of the Mexican Pacific coast until the ENSO event of 1997/98, which caused a massive mortality and the loss of near 96% of coral cover^52^. This area harbors the archipelago islas Marías, that consists of four islands: María Madre, María Magdalena, María Cleofas and San Juanito; with María Cleofas having the largest coral cover^14^. In the north (Gulf of California) we can find coral reefs in Baja California Sur State, which harbors important coral reef formations in San Lorenzo, San Gabriel and Caleritas. Until the decade of the 90 s, the coral reef of Cabo Pulmo reached more than 150 ha, and it was acknowledged as the most important in the Mexican Pacific coast, but it lost most of its coverage after the El Niño 1997^53^. Finally, the archipelago de Revillagigedo far away of the mainland, is a group of four volcanic Islands, with Socorro as the main one, which maintains a well-preserved reef in Playa Blanca, formed by branching corals of the genus *Pocillopora*, and massive corals of the genus *Porites*^54^.

### Qualitative sampling

All studied ETP reefs were between 1 and 6 m depth. In each one, the sampling was undertaken by a 2 h random dive^55^, during which three divers searched for sponges in different areas of the reef, both exposed and cryptic habitats, which included the lower surfaces of live or dead corals, interstices of coral framework, and loose heads which were overturned and examined.

Fragments of specimens that we were not able to identify “*in situ*” were collected, fixed and preserved in 70% ethanol. Spicules were cleaned in boiling nitric acid followed by water rinse and dehydration in alcohol, then dried on a microscope slide or circular cover slip for SEM (scanning electron microscopy). Spicule measurements (30 for each type) were made by light microscopy.

Sampling in cryptic spaces is difficult and time consuming, and the differentiation of individuals of sponges to estimate biomass is virtually impossible. However, the simple presence/absence is enough for species richness^56–58^ Therefore, similarity among reefs was established by means of a classification analysis, using species as variables. The similarity matrix for the classification was calculated by means of the Sørensen index based on presence/absence^59^. The results were then graphically described using dendrograms with the UPGMA (unweighted pair-group method using centroids) aggregation algorithm^60^.

**Figure 3:**
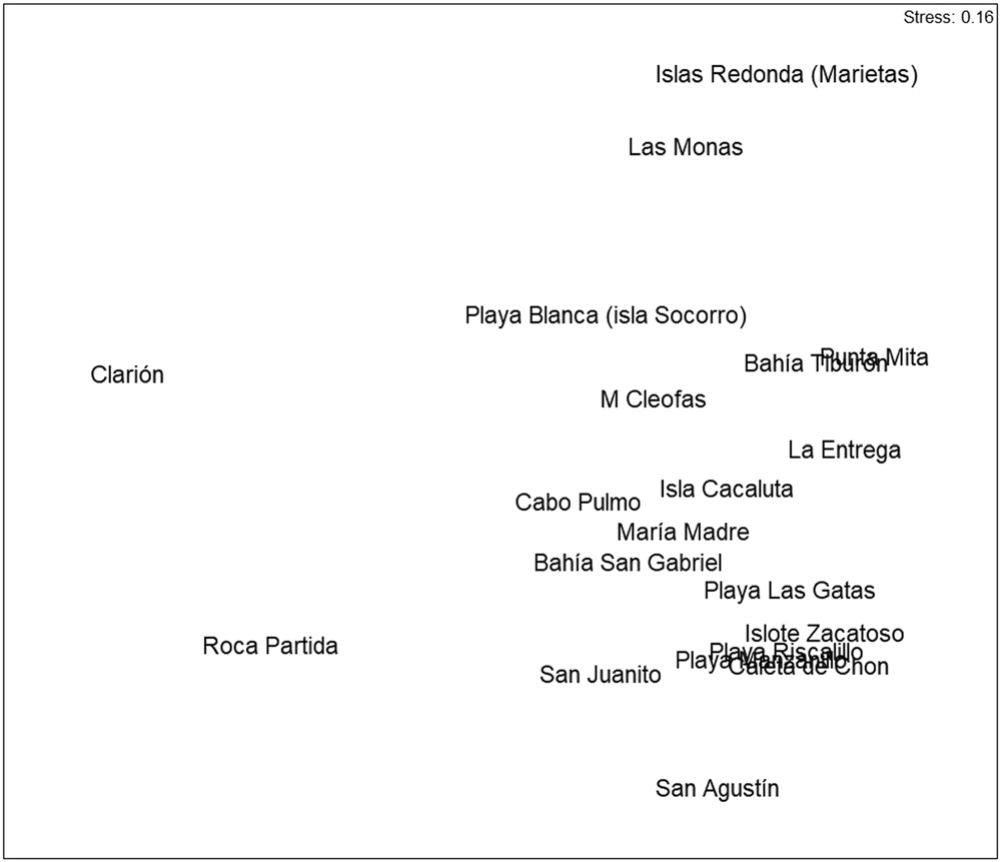
MDS ordination of the reefs using data on sponge richness.

Multidimensional scaling and ordination were used to detect community patterns, using the PRIMER (v 6.1.11) software program^61^, and a two-dimensional non-metric Multidimensional Scaling (MDS), based on the Sørensen similarity matrix, was used to visualize community patterns (Fig. 3). The adequacy of the MDS was assessed through the stress coefficient, which should be <0.15 in order to minimize misinterpretations^61^.

### Quantitative sampling

A quantitative sampling (density and species per square meter) was undertaken only in two reefs, because is a very difficult and time-consuming process. Besides, it’s also necessary brakes large coral pieces to detect cryptic sponges. However, the data can be considered as representative for the whole Mexican Pacific reefs. A previous study in Panama (Pacific side) also reached the same conclusion^62^. For that, five transects 18 m long were set up, and six quadrats of 1 m^2^ were placed along each one, resulting in a sampling area of 6 m^2^ per transect (a total of 30 m^2^ per reef). Density (ind. m^−2^) was estimated by counting all patches found inside each quadrant and later average per square meter. Species richness was estimated in the total of the sampling area (30 m^2^), and later average by square meter. In the case of boring sponges, their appearance in the samples was quantified as a unique patch due to the difficulty to differentiate among individuals.

### Data from Caribbean (CR) and West Pacific reefs (WPR)

**Figure 4:**
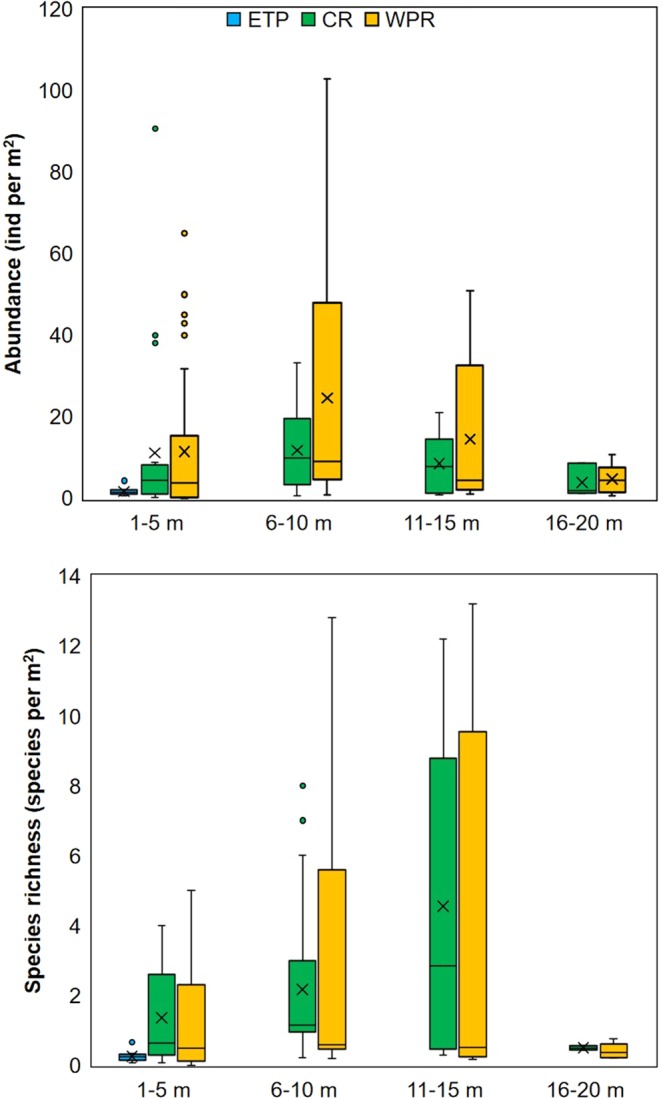
Box and whisker plot of the variation of the abundance of sponges per region (ETP, CR, and WPR), and depth (above), and species per square meter (below). Average is represented by X, median by the horizontal line inside the box. The maximum and minimum values are displayed with vertical lines (“whiskers”) connecting the points to the box. Points are outliers’ values.

In order to compare the information gained from this study with those from CR and WPR, an exhaustive research of the literature was done. All the papers about coral reef sponges with information about number of species, abundance (density), diversity (species per surface unit) were utilized to obtain mean values per basin and depth (see for example Fig. 4).

### Statistics analyses

Differences in abundance and species between different depths and regions were analyzed by two-way ANOVA after verifying normality (Kolmogorov-Smirnov test)^63^ and variance homogeneity (Levene’s test)^64^. If the results of the ANOVA’s revealed a significant difference, a post hoc analysis (Multiple Range Tests) was then performed to evaluate the differences observed. The level of significance was 5% (p < 0.05). When heteroscedasticity was detected^64^, even after transformation, a significance value of p < 0.01 was adopted to avoid Type I error. As mean values were used for each region and depth, there were no replicates that allowed for the estimation of region x depth interaction. For example, there is no data for ETP below 6 m depth, and comparison at deepest depth is not possible.

## Results

A total of 87 species belonged to 48 genera were identified. The order Clionaida had the highest number of species (23%), followed by Poecilosclerida (18%), Haplosclerida (13%) and Tetractinellida (10%). The rest of species are distributed in Agelasida, Axinellida, Bubarida, Dendroceratida, Dictyoceratida, Scopalinida, Suberitida, Tethyida and Verongida.

The most diverse family was Clionaidae, which contained four genera and 18 species, and the most common genus was *Cliona* with 13 species. In Haplosclerida the most common genus was *Haliclona* with five species, in Tetractinellida *Thoosa* with four species, and in Poecilosclerida it was *Mycale* with three species (Table 1). The species not identified are potential new species and are currently under study.

A high percentage of species (≈40%) (35 species) was recorded from one reef only, and only four species were common, co‐occurring in at least 10 different reefs (≥50% level of sites); these were *Cliona vermifera* (75% of the reefs), *Thoosa mismalolli* (63%), *Pione carpenteri* (55%) and *Cliona tropicalis* (52%). Other common species were *Siphonodictyon crypticum* (45%), *Thoosa calpulli* (45%), *Callyspongia californica* (41%) and *Mycale cecilia* (37%).

The number of species was highly variable among reefs (Table 1). The highest number was found at Playa Blanca (28 spp) (Isla Socorro, Revillagidedo archipelago), Isla Redonda (Marietas islands) (27), and Isla María Cleofas (Marías archipelago) (22); the lowest number of species was recorded at Roca Partida (4) and Clarion (5), both part of the Revillagigedo archipelago.

There were no clear groups because reefs were mixed on the cluster analysis (cluster not figured). In agreement with cluster, MDS did also not show a clear gradient and the reefs were arranged regionally only partially (stress 0.16) (Fig. 3); for example, the sponge community of Playa Blanca (Isla Socorro), is close to that of Punta Mita, although they are geographically separated from each other. On the right side of the ordination, some reefs that appeared together, as for example Islote Zacatoso, Playa las Gatas and Caleta de Chon, are spatially next to each other. In the left corner, appears some locations from Revillagigedo archipelago such as Clarion and Roca Partida, which are close to each other, and presented the lowest sponge diversity.

Regarding the quantitative sampling, the abundance varied from 0.57 to 4.3 (1.69 in average) ind. m^−2^. The number of species per m^2^ varied from 0.06 to 0.66 species per m^2^ (0.25 in average) (Fig. 4).

**Figure 5:**
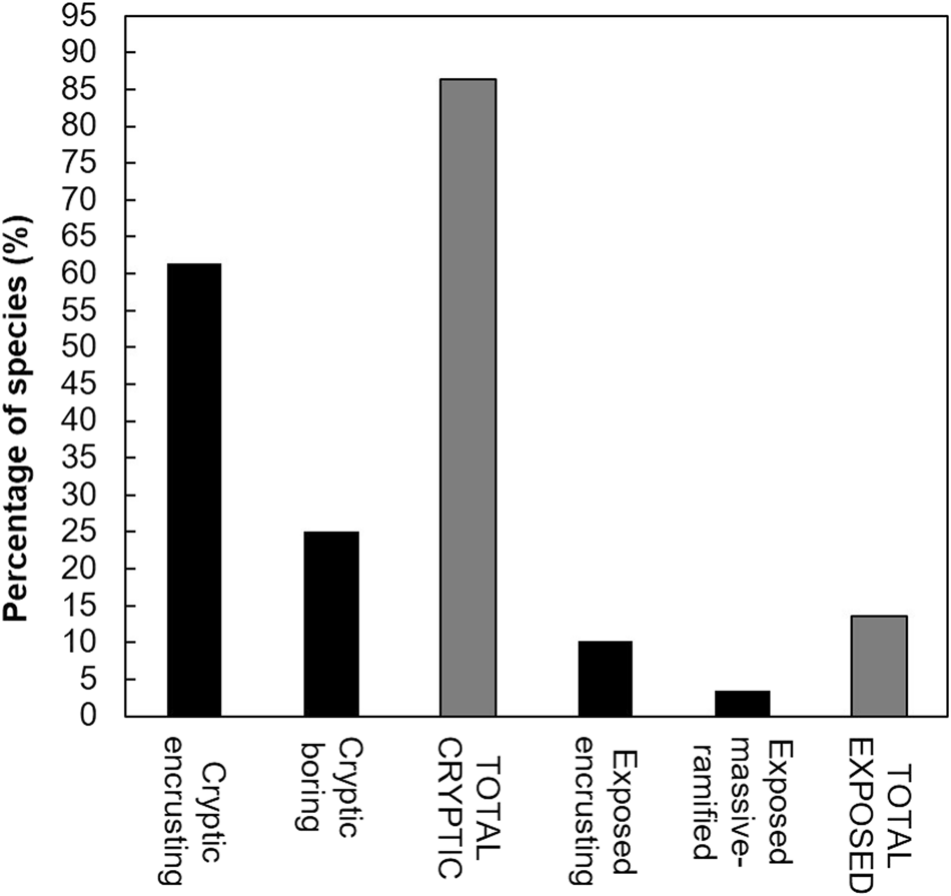
Percentage of cryptic (encrusting and boring) and exposed species in the studied ETP reefs.

**Figure 6:**
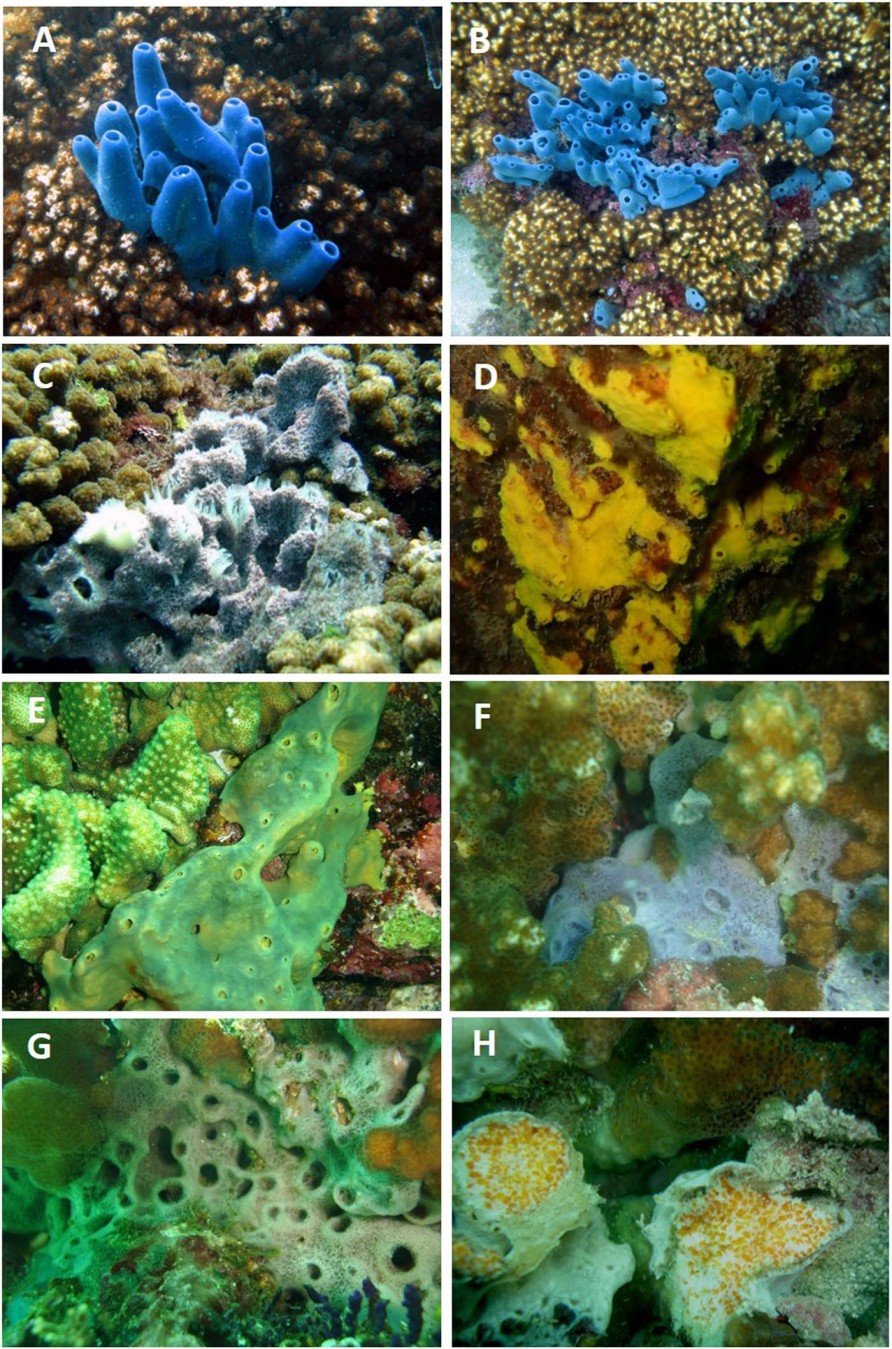
Different species found in ETP reefs. (**A**,**B**) *Amphimedon texotli*, (**C**) *Haliclona caerulea*, (**D**) *Suberea etienei*, (**E**) *Aplysina revillagegedi*, (**F**,**G**) *Callyspongia californica*. (**H**) *Cliona vermifera* boring *Pocillopora* sp. Images (**A**,**B**) taken by JACB, the rest taken by JLC.

The overwhelming majority of the species was cryptic (Fig. 5), occurring as small encrusting patches underneath coral rubbles and dead corals, or boring, measuring in the order of centimeters, only six species were relatively large measuring in the order of decimeters. The latter have the capacity of overgrowing live coral: *Callyspongia californica*, *Chalinula nematifera*, *Mycale cecilia*, *M. magnirhaphidifera*, *Haliclona caerulea*, and *Amphimedon texotli*. The last two, are the only massive species in all the reefs (Fig. 6).

It’s also important to note the rare occurrence of keratose sponges. Except for four species of *Aplysina* and one of *Ircinia*, most of the reefs, and indeed the surrounded areas, are devoid of horny species or, if they are present, they are very small.

## Discussion

Coral reefs are the largest structures created by any group of animals in the world. Their three-dimensional framework forms numerous habitats which are densely populated by an enormous variety of organisms^65^ such as sponges, which represent the major trophic link in organic matter transfer from the pelagic to the benthic compartment in these ecosystems^30,31,66^.

**Figure 7:**
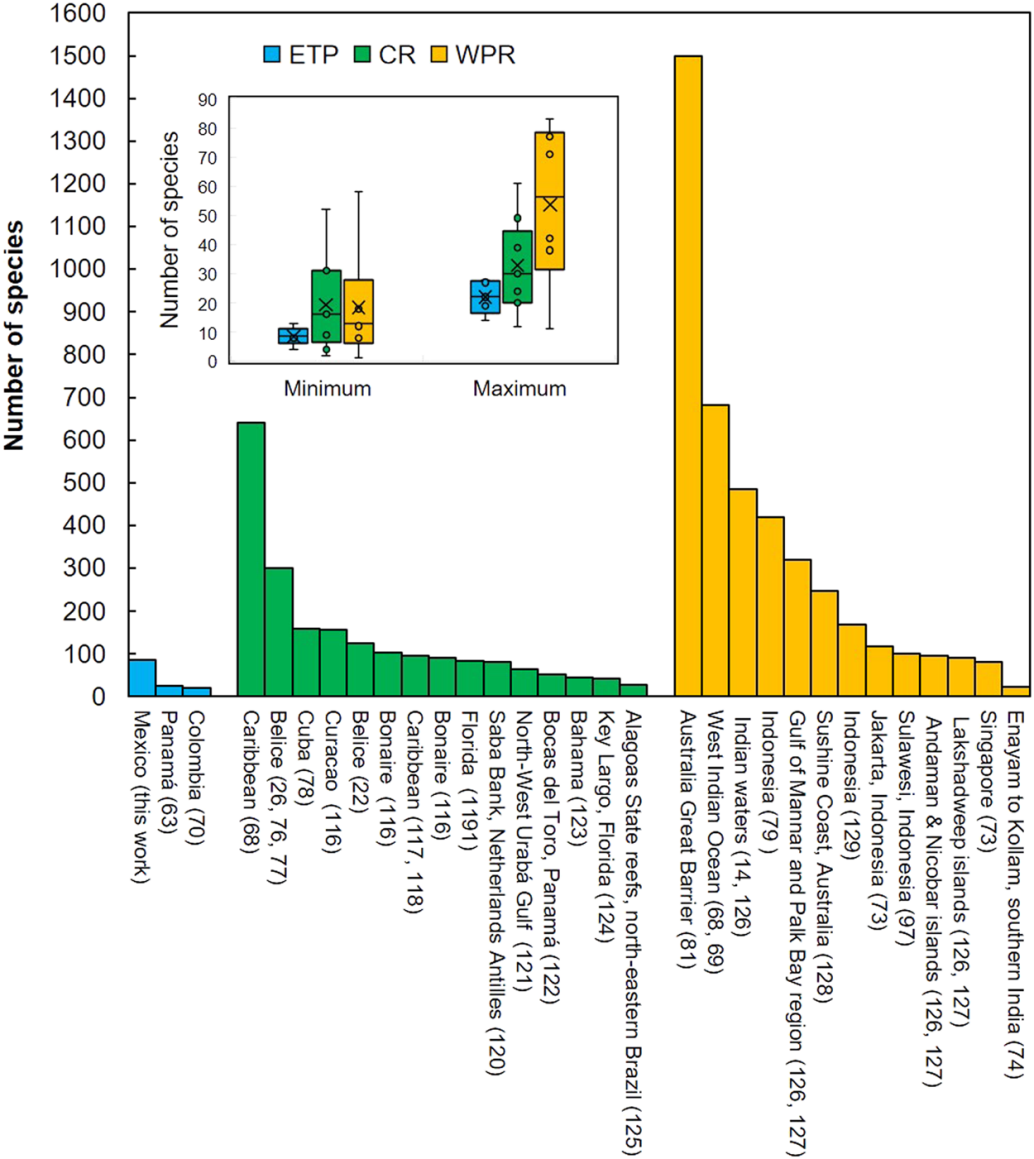
Number of total of species of sponges in different coral reefs in ETP, CR, and WPR. References are given in parenthesis. Inset: i mean of the minimum and maximum number of species per reefs per region found in the literature.

There are not many studies about diversity of sponges on coral reefs, most published papers focused on Caribbean (CR), and West Pacific (WPR)^67^, while ETP is practically unknown^68^. Previous to this study, we know only two works, one on coral reef sponges in Panama, which cited 22 species^62^, and other one from Colombia, which did not deal exclusively with coral reef sponges but other habitats as well, it recorded 21 species^69^. Thus, the present work is the first large-scale study devoted exclusively to coral reef sponges from ETP, which, despite of the high number of reefs studied, and the vast geographical area that they represent, showed a surprising low number of species (87 species). This difference is more evident if we compare the total diversity in the entire Mexican Pacific coast, with particular reefs from CR or WPR; e.g. in Thousands Islands reefs (North Western of Java), 118 species are reported^70^, in the Spermonde Archipelago (south western Sulawesi, Indonesia), 151 species are recorded^71,72^. Reefs in the Gulf of Mannar and Palk Bay region (India) harbor more than 319 species^73^ (see Fig. 7)^74–87^. A similar situation is found in the Caribbean. To quote some examples; 92 species in Bonaire reefs^88^, 124 species in Belize reefs -counting only cryptic species-^22^, which reach more than 300 species when included also exposed one^26,89,90^, 156 species in Curaçao (Saba Bank), 160 species in Cuba^91^ (Fig. 7). It is important to note that small cryptic, boring, and thinly encrusting (<4 cm in diameter) specimens were excluded from most of these studies, so, the inclusion of those, would increase dramatically the number of sponge species in CR and WPR.

By regions, the differences are more impressive yet: 420 species in coral reefs from Indonesia (830 in total in the country)^92^, 486 in coral reefs from Indian waters^73,92,93^, or 1500 species for the Great Barrier from Australia^94^. The WPR, particularly the “coral triangle” region, support the most diverse sponge assemblages in the world, probably including a high number of yet undescribed species.

When we compared standardized measures, such as abundance and species per square meter, the difference was also remarkable, with ETP drastically much lower than others regions (Fig. 4). In ETP we have 1.6 ind. per m^2^ and 0.25 species per m^2^, in average. At a similar depth (below 6 m), CR and WPR have seven times more ind. per m^2^ (≈11 ind. per m^2^), and between four (1.3 for CR) and five times (1.4 for WPR) more species per m^2^. These differences between ETP and the other two basins were significant (Tables 2 and 3).

**Table 2:** Summary of the two-way ANOVA for differences in abundance (ind. per m^2^) in ETP, CR and WPR, at different depths (see Fig. 4).

Source	Sum of Squares	Df	Mean Square	F-Ratio	P-Value
**MAIN EFFECTS**					
Region	5008.84	2	2504.42	7.17	**0.0010**
Depth	3804.32	3	1268.11	3.63	**0.0141**
RESIDUAL	61088.5	175	349.077		
TOTAL (CORRECTED)	69988.7	180			
**Multiple Range Tests for Abundance**					
	**Count**	**LS Mean**	**LS Sigma**		
ETP	23	−2.44891	4.28989		
CR	54	8.11227	2.83822		
WPR	104	13.5795	2.25136		
**Contrast**	**Difference**	+/− **Límits**			
**1–5 m ETP vs 1–5 m CR**	10.5612	9.30228			
**1–5 m ETP vs 1–5 m WPR**	−16.0285	8.57705			
**6–10 m CR vs 11–15 m WPR**	15.5055	13.1302			

Bold denotes a statistically significant difference. All F-ratios are based on the residual mean square error.

**Table 3:** Summary of the two-way ANOVA for differences in number of species in ETP, CR and WPR, at different depths (see Fig. 4).

Source	Sum of Squares	Df	Mean Square	F-Ratio	P-value
**MAIN EFFECTS**					
Region	79.234	2	39.617	4.58	**0.0116**
Depth	99.6681	3	33.222	3.84	**0.0109**
RESIDUAL	1419.26	164	8.654		
TOTAL (CORRECTED)	1593.38	169			
**Multiple Range Tests for Species**					
	**Count**	**LS Mean**	**LS Sigma**		
ETP	26	0.226354	0.588949		
CR	46	1.89334	0.476498		
WPR	98	2.2271	0.357542		
**Contrast**	**Difference**	+/− **Límits**			
1–5 m ETP vs 1–5 m CR	1.66764	1.44408			
**1–5 m ETP vs 1–5 m WPR**	−2.00136	1.30785			

Bold denotes a statistically significant difference. All F-ratios are based on the residual mean square error.

Comparison at deepest depth is not possible because there is no data for ETP below 6 m depth. The difference between CR and WPR was only significant for abundance at the 6–10 m depth interval (highest values): 12 vs 22 ind. per m^2^; respectively. Previous studies also showed that diversity (per unit-area) is similar in CR and WPR, but sponge biomass is greater in CR^95,96^. The decrease of diversity at 16–20 m is better explained in terms of the smaller number of papers that report information at this depth, rather than an inherently poor fauna. This increase of abundance (density and cover) along a depth gradient with highest values at intermediate depths seems to be a general pattern of coral reef sponges previously observed in CR^97,98^.

Explaining the differences between CR and WPR is beyond the goal of this research. However, previous studies showed that factors such as food limitation^99,100^, chemical defense^101^, and nutritional strategies, with CR sponges more heterotrophic, and WPR more autotrophic^95,96^ could be responsible of the differences.

In ETP as in CR^72,102,103^ [among others] and WPR^99,100^ [among others] very few species dominate the sponge assemblages, with a high percentage of species recorded from only a single site. This seems to be a general pattern in coral reef sponges worldwide. However, in ETP, the species that dominate the assemblages are boring sponges such as *Cliona vermifera, Thoosa mismalolli* or *Pione carpenteri*, which have a wide ETP occurrence and very broad ecological distribution. The prevalence of boring sponges in Mexican reefs is very interesting and remarkable, since these sponges are highly resilient to anomalous temperature shifts^104,105^, especially when compared to tolerance thresholds found for corals^106,107^. Previous studies showed that high anomalous temperatures that were detrimental to corals, had no negative effect in abundance and reproduction of *C. vermifera*^108^. The resilience demonstrated by boring sponge species in the ETP to thermal shock supports ecological projections that sponges will become an increasing threat to coral and coral reef health. However, recently it has been shown that elevated temperature can disrupt the functionality of microbial symbionts of *Cliona orientalis*, which occurred at a lower temperature than the 32 °C threshold that induced sponge bleaching^109^.

In summary, ETP coral sponges are not only less diverse compares to CR and WPR, there are also striking differences in growth form and size, because they are mostly cryptic encrusting, and very small in size (generally less than a few square centimeters). No sponges can be seen, except by close inspection of the bases of corals or by breaking open the reef frame (Fig. 2). In contrast, in CR and WPR sponges are more diverse, also in morphology, more species are living on exposed surfaces, they reach larger sizes, to over 2 m in largest dimension, and they can constitute the most abundant animals on the reef. For example, in the Wakatobi Marine National Park (Sulawesi, Indonesia) more than 200 individuals per m^2^ have been reported which occupy more space than the corals^110^. Similar to CR, in WPR many large, conspicuous sponges are present, such as the giant barrel sponge *Xestospongia testudinaria* or species of *Lamellodysidea, Phyllospongia*, and *Carteriospongia*.

### Explaining the interoceanic differences: the influence of local and large-scale factors

Different theories have arisen to explain the pattern observed in ETP, particularly considering the dominance of encrusting and cryptic species, and the low diversity of their assemblages^111^. Prevalence of small cryptic encrusting sponges has been traditionally explained by the predation pressure by spongivorous fishes^112^, since cavities provide some advantage to cryptobionts by excluding certain predators. However, the pressure from spongivores is a common factor in the three areas^100^ for WPR^101,113,114^ [among others for CR and^115,116^ for ETP]. There are also other sponge predators than fishes such as mollusks, echinoderms, and crustaceans, all of which have cryptobiontic representatives^117–119^.

Perturbation, at local and large scale, rather than biologicals factors, seems to explain the low prevalence and characteristics of sponge assemblages in ETP reefs, which are very frequently located in shallow water, where turbulence is periodically very strong, which, together with abrasion provoked by particles in suspension, sedimentation^41,120^, and high levels of damaging light, limit sponge survival and shape ETP sponge assemblages. The low sponge abundance at shallow depth in the Caribbean too has been associated with turbulence and numerous studies^97–100,112,121^, among others concluded that three depth-related factors influence sponge community structure on most Caribbean reefs: turbulence, spatial competition and predation. The first two only influence sponge communities at shallow depths, mostly above 10 m, and competition mostly above 20 m. In WPR, the exclusion of sponges from shallow waters was also attributed to excessive turbulence and possibly by high levels of damaging light^28,70,122^ among others. Exposed reefs at Isla del Coco^123^, and Clipperton^124^, which present a similar pattern with a high proportion of thin encrustations, support the suggestion that the turbulence and abrasion shape shallow coral reef sponge communities.

Beside the influence of local abiotic variables that could explain the low diversity and the prevalence of small encrusting species in ETP reefs, and indeed in CR and WPR shallow reefs, it is important to highlight the recurrent large-scale phenomena in the ETP, such as frequent upwellings that bring cold water up onto the reefs^12^, and periods of high-water temperature during El Niño years, which cause death and destruction of corals that have not had the chance to reach the levels of development found in the CR^15^. Moreover, ocean conditions in CR are relatively constant providing an environment that is conducive to reef growth (the average age of Caribbean reefs is 5600 years old). ETP reefs are smaller, younger (varying from 200 to 5600 years old), and with variable conditions where disturbances are more pronounced^125^.

### Explaining the interoceanic differences: the influence of evolutionary history

A hypothesis, which serves to explain the impoverished nature of the ETP coral fauna, is based on the unstable composition of faunas in remote marginal regions, and in the low resilience of these faunas. Due mainly to physical perturbations commented above, species already living near their tolerance limits become locally extinct and are not soon replenished after disturbances because of their isolation from source populations^126^.

The separation of ETP and the CR, occurred 3.5 million years ago, stopping the flow of species from the CR to the ETP, and since then, ETP has been highly isolated by cool currents from the north and south, and the Eastern Pacific Barrier (EPB) to the west; a vast expanse of deep water^16,125^. The isolation of the ETP sponge assemblages is supported by the fact that sponge fauna of Clipperton Island has stronger affinities with the Central and West Pacific regions than with the East Pacific region with which it shares only two or three species^124^. The majority of Clipperton species appears to have invaded from the west, evidenced by shared distributions or occurrence of close relatives in Hawaii, Tuvalu, Indonesia, New Caledonia and Australia. A study of the corals of Clipperton^127^ came to a similar conclusion.

High-diversity locations such as the Philippines, Indonesia, or the Great Barrier Reef show a greater resilience to recurrent disturbances^128–131^ that depauperate, marginal sites [e.g., the Galapagos Islands, Panama, Hawaii^7,132^, and indeed, a larger capacity to recovery after disturbances. They are also evidences that show that the cryptobenthic fauna of the Gulf of California is highly vulnerable to natural and anthropogenic disturbance as a result of the high specificity in habitat use of dominant species, and its low diversity, which limits the potential functional redundancy of the system, compromising the ecosystem’s functioning, resilience and stability^133^.

Unequal rates of speciation, extinction and migration have resulted in greater diversity in the Caribbean than in the Pacific since, ETP reefs are also impoverished with respect to coral diversity (130 species in ETP vs 240 in CR), gorgonians, zoanthids, calcareous green algae, and other sessile groups^111,134,135^.

All this, suggests that current patterns of biodiversity should be interpreted in light of both contemporary and historical processes, which have been hypothesized to be most important for taxonomic groups with poor dispersal abilities^128^.

In conclusion, factors such as isolation, difficulty to gain recruits from distant areas, perturbation, resilience, age of the reef, allowed processes like natural selection to change the species composition of each area^12^.
